# Computational Model of Calcium Signaling in Cardiac Atrial Cells at the Submicron Scale

**DOI:** 10.3389/fphys.2018.01760

**Published:** 2018-12-10

**Authors:** Miquel Marchena, Blas Echebarria

**Affiliations:** Departament de Física, Universitat Politècnica de Catalunya, Barcelona, Spain

**Keywords:** calcium modeling, atrial cells, local calcium signaling, calcium release unit, ryanodine receptor

## Abstract

In cardiac cells, calcium is the mediator of excitation-contraction coupling. Dysfunctions in calcium handling have been identified as the origin of some cardiac arrhythmias. In the particular case of atrial myocytes, recent available experimental data has found links between these dysfunctions and structural changes in the calcium handling machinery (ryanodine cluster size and distribution, t-tubular network, etc). To address this issue, we have developed a computational model of an atrial myocyte that takes into account the detailed intracellular structure. The homogenized macroscopic behavior is described with a two-concentration field model, using effective diffusion coefficients of calcium in the sarcoplasmic reticulum (SR) and in the cytoplasm. The model reproduces the right calcium transients and dependence with pacing frequency. Under basal conditions, the calcium rise is mostly restricted to the periphery of the cell, with a large concentration ratio between the periphery and the interior. We have then studied the dependence of the speed of the calcium wave on cytosolic and SR diffusion coefficients, finding an almost linear relation with the former, in agreement with a diffusive and fire mechanism of propagation, and little dependence on the latter. Finally, we have studied the effect of a change in RyR cluster microstructure. We find that, under resting conditions, the spark frequency decreases slightly with RyR cluster spatial dispersion, but markedly increases when the RyRs are distributed in clusters of larger size, stressing the importance of RyR cluster organization to understand atrial arrhythmias, as recent experimental results suggest (Macquaide et al., 2015).

## 1. Introduction

Calcium is one of the most important intracellular messengers, and thus the mechanisms that control the intracellular free calcium concentration are of fundamental physiological importance (Berridge, 1997). For instance, Ca^2+^ takes part in oocyte activation at fertilization (Poenie et al., 1985), axonal growth (Bixby and Harris, 1991), cell migration (Huttenlocher et al., 1997), gene expression (Bading et al., 1993), formation of nodules in plant root hairs (Ehrhardt et al., 1996), development of muscle (Ford and Podolsky, 1972), release of cytokines from epithelial cells (Kaufman and Roizman, 1989), cell death (Schanne et al., 1979; Farber, 1981), and excitation-contraction coupling in muscle cells (Fabiato and Fabiato, 1979).

In cardiac cells, calcium dysregulation has been related to the appearance of arrhythmias and sudden cardiac death. A life-threatening arrhythmia, fibrillation, results when an electrical wavebreak induces reentry and triggers a cascade of new wavebreaks. Ventricular fibrillation (VF) is the most common cause of sudden death, whereas atrial fibrillation (AF), the most prevalent clinical arrhythmia, accounts for nearly one third of strokes in the elderly (Weiss et al., 2005). Clinically, AF duplicates the mortality rate and increases the risk of ictus (in which poor blood flow to the brain results in cell death) 5-fold. In spite of this the treatment of AF remains deficient or inefficient, because of the incomplete knowledge of the complex pathophysiology of this disease. Often, AF has been linked to a dysregulation in the dynamics of intracellular calcium, thus the importance of a good knowledge of calcium handling dynamics in the cell. On the other hand, in the last ten years, the refinement of the experimental techniques, such as STED and dSTORM (Hell and Wichmann, 1994; Izu et al., 2006; Soeller and Baddeley, 2013) has provided, for instance, a link between the calcium handling microstructure and the occurrence of cardiac diseases, as AF (Macquaide et al., 2015), prompting the quest for more detailed models of calcium handling, able to mechanistically explain this relation.

**Figure 1:**
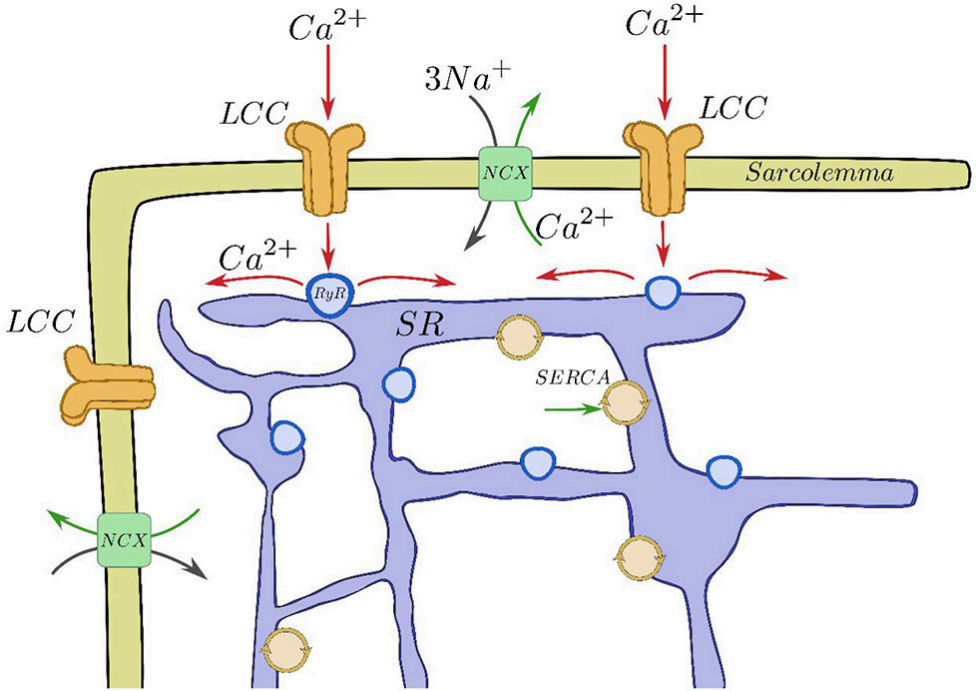
Basic components of the CICR process. Calcium enters through the LCCs, stimulating release from the RyRs, that then is reuptaken into the SR by SERCA and taken out of the cell by the sodium-calcium exchanger.

Inside cardiac cells, most intracellular calcium is stored in a complex structure called sarcoplasmic reticulum (SR), see Figure 1. Ca^2+^ is released from this internal network via the Ryanodine Receptors (RyR, Franzini-Armstrong and Protasi, 1997) when a threshold calcium concentration in the cytoplasm is achieved. This happens due to a small influx of calcium through the L-type calcium channels (LCC) during the cardiac action potential. This current triggers calcium release from the SR by activating the RyRs. RyRs open and close collectively in clusters forming functional units known as Calcium Release Units (CaRU), which are often confronted to a cluster of LCCs. In each CaRU the number of RyR and LCC is small (of the order of 10–100 of the former and 5–10 of the latter), thus its dynamics is intrinsically stochastic. CaRUs are distributed inside the cell, resulting in random and discrete Ca^2+^ release events, known as Ca^2+^ sparks (Cheng et al., 1994). A Ca^2+^ spark has been considered as the unitary dynamical element which produces the cellular Ca^2+^ dynamics, such as Ca^2+^ waves and oscillations (Falcke, 2003). The (seemingly deterministic) global calcium signal appears from the coordination of several tens of thousands of these CaRUs.

After the excitation process, Ca^2+^ removal allows relaxation of the cardiac muscle. This requires Ca^2+^ transport out of the cytoplasm by several pathways. The concentration in the SR is recovered by the active pumping of calcium from the cytoplasm to the SR carried out by the Sarcoplasmic Reticulum Ca^2+^-ATPase (SERCA). Moreover, the Na-Ca exchanger pumps Ca^2+^ out of the cell. The whole process described is called calcium-induced calcium release (CICR, Berridge, 1993; Clapham, 1995). CaRUs not just couple SR and cytoplasm Ca^2+^ concentrations via Ca^2+^ release but they are also correlated due to the Ca^2+^ diffusion in both domains. Therefore, the behavior of a single CaRU depends on the behaviors of the neighboring CaRUs.

Even though the same mechanism (CICR) triggers the transient elevation of Ca^2+^ in both ventricular and atrial myocytes, there are substantial differences in the intracellular structures. The absence of transversal tubules (t-tubules) in atrial myocytes produces inhomogeneous spatio-temporal calcium patterns when the CICR occurs. In particular, the excitation starts at the cell membrane and then propagates inward, resulting in a delay in activation time between the subsarcolemma and the cell interior. This is a key difference between atrial and ventricular cells. In the latter, the opening of LCC channels along the t-tubules triggers the release of calcium from the SR, resulting in a homogenized calcium pattern. In the former, this trigger is due to the inward wave.

Detailed models of calcium handling have been first developed for ventricular cells, including the stochastic modeling of each individual CaRU, coupled then by diffusion. In this framework, each CaRU is typically divided into different subcompartments, in which the calcium concentration is assumed to be homogeneous (Restrepo et al., 2008; Rovetti et al., 2010), although some recent models consider also calcium diffusion within the CaRU (Nivala M. et al., 2012). These models have been very successful in reproducing several calcium dysfunctions, such as calcium alternans (Restrepo et al., 2008; Rovetti et al., 2010; Alvarez-Lacalle et al., 2015) or spontaneous calcium release induced delayed afterdepolarizations (Song et al., 2015). Current advances in microscopy have allowed the development of very detailed models of calcium release at the level of the CaRU, including realistic shape of the SR, the RyR cluster, myofibrils and the mitochondria (Kekenes-Huskey et al., 2012; Hatano et al., 2013; Hake et al., 2014; Rajagopal et al., 2015).

Modeling is less developed for the case of atrial cells (Heijman et al., 2016). Common pool models, in which calcium concentration is considered to be homogeneous in each of several compartments (SR, cytosol, dyadic space, etc) have been developed for rabbit (Lindblad et al., 1996), dog (Ramirez et al., 2000), mouse (Davies et al., 2014), and human (Courtemanche et al., 1998; Nygren et al., 1998; Grandi et al., 2011; Lugo et al., 2014). One of the first models that took into account inward wave propagation was by Koivumäki et al. (2011), where the bulk cytoplasm and SR spaces were divided into several compartments, being thus a one-dimensional model, allowing for centripetal but not lateral diffusion. A similar model was also used by Li et al. (2012), showing the presence of alternans. A model allowing for both centripetal and lateral diffusion, as well as stochastic RyR gating was developed by Voigt et al. (2013), in order to study the mechanisms of after-depolarizations and triggered activity in paroxysmal atrial fibrillation. Calcium wave initiation and propagation has been considered by Thul et al. (2012) in a three-dimensional geometry, assuming a diffuse and fire model for calcium release. Finally, Macquaide et al. (2015) developed a detailed three-dimensional bidomain model of calcium propagation to study intra-CaRU cluster interactions, supporting the idea that cluster fragmentation and redistribution sustains atrial fibrillation through the enhancement of calcium release.

Still, there are several open questions regarding CICR in atrial cells. To name some: (1) the role of buffers, RyR sensitivity and the level of cytosolic calcium in calcium wave propagation; (2) the effect of the RyR cluster spatial structure and size distribution; (3) the role of t-tubules (if present). Most subcellular ventricular and atrial models (Restrepo et al., 2008; Rovetti et al., 2010; Voigt et al., 2013) consider the cell divided into several thousands of functional units (CaRUs). Each CaRU is then divided into different compartments, replicating at the subcellular scale the structure of common pool models. Despite the success of such models to replicate calcium transients and spark characteristics, they are not well-suited to study the effects of changes in the microstructure (position of the RyR clusters, inhomogeneities, etc). Rather, to study the effect of RyR cluster distribution on wave propagation, continuum models of calcium diffusion with point release sites have been considered, although often with simplified release dynamics (Izu et al., 2006; Thul et al., 2012, 2015; Øyehaug et al., 2013). On the other hand, very detailed models at the level of the CaRU (Hake et al., 2014) are very demanding computationally, and typically not well-suited to study effects that require of long simulation times, as calcium homeostasis or spark rates. With that in mind, we present a subcellular calcium atrial model where the homogenized local behavior is described with a two-concentration field model, using effective diffusion coefficients of calcium in the SR and in the cytoplasm, with stochastic gating of the RyRs and LCCs. This model follows the spirit of earlier bidomain models (Jafri and Keizer, 1995; Keener and Sneyd, 1998), defining at each point in space cytosolic and SR calcium concentrations, with given volume fractions (Keizer and De Young, 1992). The model presents some important characteristics: (1) a very fine discretization, making it possible to describe (even if coarsely) the RyR cluster structure; (2) incorporation of the cell structure with distinction between z-lines and normal cytosol in terms of the volume ratio of SR and cytosolic volumes, diffusion constants and presence of buffers; (3) freedom to set the center of the RyR clusters arbitrarily, that do not need to be disposed in an homogeneous regular grid. In this paper, we focus on the effect of CaRU spatial structure and distribution, and find that a more disordered distribution of the CaRUs presents a lower frequency of sparks in resting conditions. On the contrary, when the spatial distribution is maintained constant, but the RyRs are distributed in a smaller number of larger CaRUs (so the total number of RyRs remains constant), the spark frequency increases, in accordance with experimental results in cells presenting AF (Macquaide et al., 2015).

## 2. Methods

Our computational model performs single cell simulations and is based on homogenization (Goel et al., 2006). Although it is well-known that the SR forms a branching network (largely interconnected), with an interior that is distinct from the cell cytoplasm, this fact has largely been ignored, with most models making the a priori assumption that a Ca^2+^ concentration for both the SR and the cytoplasm can be defined at each point in space. So that, the cytoplasm and the SR are assumed to coexist at every point in space. For this reason, a fraction of each volume is occupied by the cytoplasm (*v*_*i*_) and the complementary fraction by the SR (*v*_*sr*_), given that *v*_*i*_ + *v*_*sr*_ = 1.

We define *c*_*i*_, *c*_*sr*_, and *c*_*bi*_ as the concentration of calcium in the cytoplasm, the SR, and the concentration of calcium bound to buffers. This description assumes that there exist effective diffusion coefficients *D*_*i*_ = *D*_*i*_(*v*_*i*_) and *D*_*sr*_ = *D*_*sr*_(*v*_*sr*_) that, in an average sense, incorporate the effect of that complex geometry. Although in principle these coefficients could be calculated knowing the SR structure (Goel et al., 2006), we will take the functional forms used in Goel et al. (2006). Since both fractions, *v*_*i*_ and *v*_*sr*_, vary in different parts of the cell, it implies that both diffusion coefficients are functions of the position, *D*_*i*_ = *D*_*i*_(**r**) and *D*_*sr*_ = *D*_*sr*_(**r**). In our simulations we take the values *D*_*i*_ ~ 250 μm^2^/s and *D*_*sr*_ ~ 90 μm^2^/s, that are within the upper range considered in the literature (Louch et al., 2010; Bers and Shannon, 2013).

The cardiac cell is modeled as a two dimensional domain with *L*_*x*_ = 100 μm and *L*_*y*_ = 15 μm. The spatial grid belongs to the submicron scale and it is defined as *dx* = *dy* = 0.1μm. There are points of the grid with and without RyRs. A typical RyR has a size of 30 x 30 nm. The RyRs are transmembrane proteins located at the surface of the SR, so they form a 2D grid. Thus in each of our grid points we locate a maximum of 10 RyRs.

**Figure 2:**
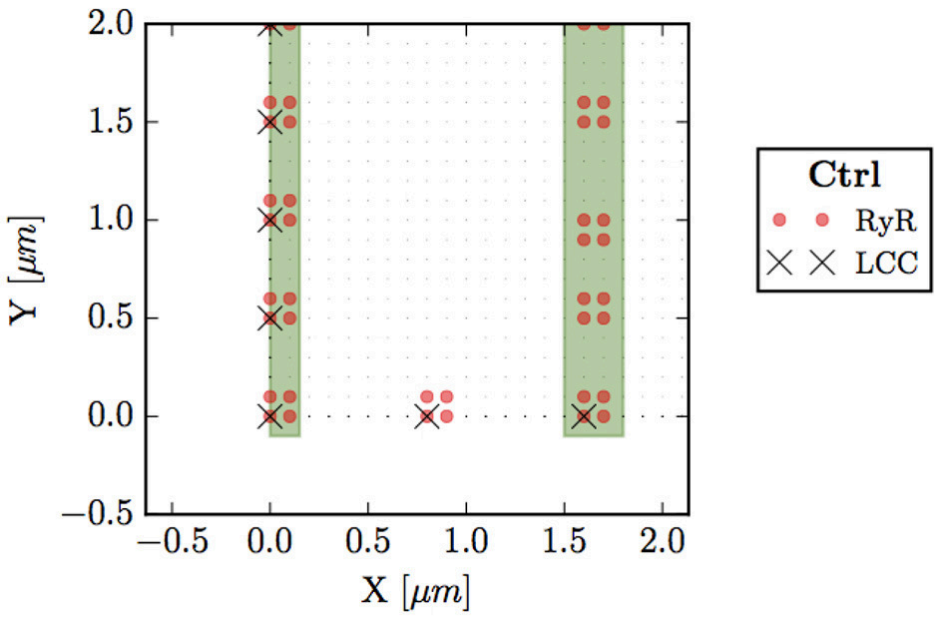
Bottom left corner of the whole cell. Circles (red) represent points with RyRs, black crosses are LCC groups, green stripes indicate the z-line region (domain Ω_*sr*_), and small dots the sarcomere (Ω_*c*_).

A collection of grid points presenting RyRs form a cluster, i.e., a CaRU. In atrial cells, CaRUs are arranged periodically in the longitudinal and transversal directions, with some—seemingly Gaussian—dispersion (Chen-Izu et al., 2006). In our model, we place the centers of the clusters on the perimeter following an exact periodic distribution with a period ${\bar{T}}_{x}={\bar{T}}_{y}=0.5$ μm (see Figure 2). In front of all these exterior CaRUs there are LCC groups. Inside the cell, CaRUs are placed following a Gaussian distribution centered at the z-lines and with a fixed dispersion σ. We take σ = 0.4 μm as standard value. The average distance between CaRUs is *T*_*x*_ = 1.6 μm and *T*_*y*_ = 0.5 μm. Experimental data shows that the SR domain coincides with these z-lines (Soeller et al., 2007). In this sense, we identify the z-lines with periodic narrow strips (0.3 μm width) with a predefined period (*T*_*x*_). Let be Ω_*c*_ the sarcomere domain, that is, the zone between z-lines and let be Ω_*sr*_ the zone contained in z-lines and all the contour (∂Ω). Notice that Ω_*c*_∩Ω_*sr*_ = ∅. Besides, we consider the presence of Ca^2+^ buffers: troponin (TnC), Calmodulin (CaM), and SR Ca-binding sites. The TnC buffer affects the cytoplasmic concentration of calcium in the Ω_*c*_ domain. The other buffers, calmodulin and SR, affect also *c*_*i*_ but in all the cell, Ω_*c*_∪Ω_*sr*_. We assume that all the buffers are immobile.

Because of the homogenization coarse grain, we define *c*_*i*_(**r**, *t*) (free calcium concentration), *c*_*sr*_(**r**, *t*) (calcium concentration in the SR), and *c*_*bi*_(**r**, *t*) (calcium attached to buffers: TnC, CaM, and SR buffer) in all points. Therefore, we state the problem with the following set of partial differential equations (PDEs).

(1) $$\begin{array}{l} \frac{\partial c_{i}\left(\text{r},t\right)}{\partial t}=J_{i}\left(\text{r},t\right)+\nabla \cdot \left[D_{i}\left(\text{r}\right)\nabla c_{i}\left(\text{r},t\right)\right]-J_{bi}\left(\text{r},t\right) \end{array}$$

(2) $$\begin{array}{l} \frac{\partial c_{sr}\left(\text{r},t\right)}{\partial t}=\frac{v_{i}\left(\text{r}\right)}{v_{sr}\left(\text{r}\right)}J_{sr}\left(\text{r},t\right)+\nabla \cdot \left[D_{sr}\left(\text{r}\right)\nabla c_{sr}\left(\text{r},t\right)\right], \end{array}$$

(3) $$\begin{array}{l} \frac{\partial c_{bi}\left(\text{r},t\right)}{\partial t}=J_{bi}\left(\text{r},t\right), \end{array}$$

where *J*_*i*_ and *J*_*sr*_ are the fluxes into the cytosol and the SR spaces, respectively, *J*_*bi*_ accounts for the binding of free calcium to the different buffers. In order to relate the fluxes between the cytoplasm and the SR we have multiplied by the volume fraction *v*_*i*_/*v*_*sr*_, that depends on **r**, that is, on the domain Ω_*c*_ and Ω_*sr*_. In addition, each point could have different components (RyR or not, LCC or not) and could belong to the membrane or not. The fluxes that may contribute to the total flux into the cytosol *J*_*i*_ are the SR release flux *J*_*rel*_, the SERCA pump *J*_*up*_, the L-type calcium flux *J*_*CaL*_ and the sodium-calcium exchanger flux *J*_*NaCa*_. The release flux *J*_*rel*_ carries Ca^2+^ ions from the SR to the cytoplasm through the RyRs. Thus, it exists only on those points that have a CaRU, indicated by a red dot in Figure 2. *J*_*up*_ pumps calcium from the cytoplasm to the SR and it is present in all cell domain (Ω_*c*_∪Ω_*sr*_). The sum of these two fluxes (when appropriate) constitute the total flux from the SR. Then, *J*_*CaL*_, the inward L-type calcium flux, depends on the LCC clusters, so that it will act on those points that contain this channel, indicated by a cross in Figure 2. Indeed, LCCs appear only in some points of the cell membrane, ∂Ω (those that also have a CaRU). Finally, the NaCa exchanger, *J*_*NaCa*_, acts along all the perimeter ∂Ω.

A detailed description of all the fluxes can be found in the Supplementary Material. Below we present some details of the release and L-type calcium fluxes.

### 2.1. Release Flux

As shown in Figure 2, we consider each CaRU formed by several grid points containing RyRs. As standard for a CaRU, we consider one containing 36 RyRs, divided equally among 4 grid points, each one containing 9 RyRs. We will change this configuration in section 3.4 to consider larger CaRUs, maintaining fixed the total number of RyRs in the cell. This resembles the situation found in cells presenting AF (Macquaide et al., 2015).

**Figure 3:**
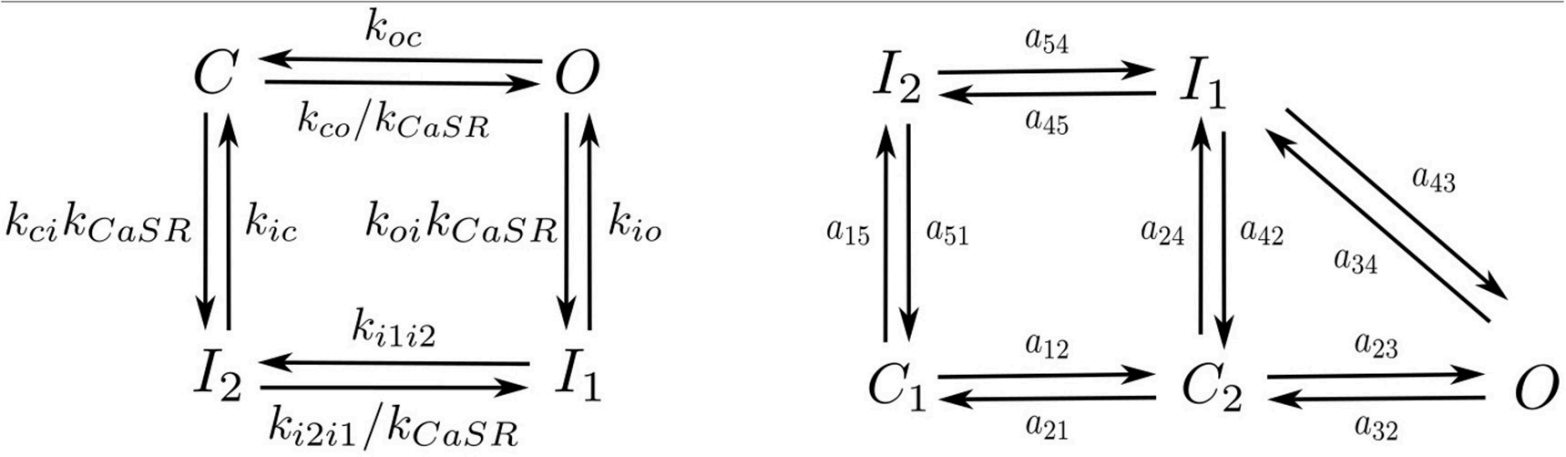
Markov models of the RyR **(Left)** and LCC channels **(Right)**.

Following Stern et al. (1999) each RyR can be in one of four different states: close *C*, open *O*, and two inactivated states *I*_1_, *I*_2_ (Figure 3). Calcium release from the SR to the cytoplasm is taken to be proportional to the concentration difference and the number of RyR in the open state, *O*_*RyR*_,

(4) $$\begin{array}{l} J_{rel}=g_{rel}O_{RyR}\left(c_{sr}-c_{i}\right). \end{array}$$

This flux is only present in those points that present RyRs (highlighted in red in Figure 2).

### 2.2. L-Type Calcium Flux

The inward current of calcium from the extracellular medium toward each CaRU is dependent on the number of LCC channels in the open state *O*_*LCC*_, the voltage, and the local calcium concentration in these points, which are close to the membrane, according to

(5) $$\begin{array}{l} I_{CaL}=g_{CaL}O_{LCC}4z_{m}\frac{e^{2z}c_{i}-{\left[Ca\right]}_{0}}{e^{2z}-1}, \end{array}$$

where *z* = *VF*/(*RT*) and *z*_*m*_ = 0.341*zF*. The current *I*_*CaL*_ is converted to the flux *J*_*CaL*_, with units of μ M/ms, through:

(6) $$\begin{array}{l} J_{CaL}=\frac{I_{CaL}}{2Fv_{myo}}, \end{array}$$

where *v*_*myo*_ is the volume of the cytosol.

We have used the LCC model described in Mahajan et al. (2008) with some changes in the parameters as in Alvarez-Lacalle et al. (2015). We consider the presence of 5 LCC channels in each CaRU (located all in the same grid point) with five possible states (Figure 3): two closed states (*C*_1_ and *C*_2_), two inactivated states (*I*_1_ and *I*_2_) and one open state (*O*). The stochastic dynamics of the transitions is implemented using a time-adaptive Gillespie's method (Nivala J. et al., 2012). The transition rates *a*_*ij*_ are described in the Supplementary Material.

### 2.3. Other Fluxes

There are extra fluxes that appear on the model. The Na-Ca exchanger and the SERCA pump are both explained in the Supplementary Material.

## 3. Results

### 3.1. Calcium Handling Characteristics

The calcium trace results from the sum of calcium at different sites. Since in our model the volume fraction changes from site to site, we have to define the average calcium concentrations as:

(7) $$\begin{array}{l} \langle c_{i}\rangle =\frac{\sum_{\text{r}}v_{i}\left(\text{r}\right)c_{i}\left(\text{r},t\right)}{\sum_{\text{r}}v_{i}\left(\text{r}\right)},\langle c_{sr}\rangle =\frac{\sum_{\text{r}}v_{sr}\left(\text{r}\right)c_{sr}\left(\text{r},t\right)}{\sum_{\text{r}}v_{sr}\left(\text{r}\right)} \end{array}$$

**Figure 4:**
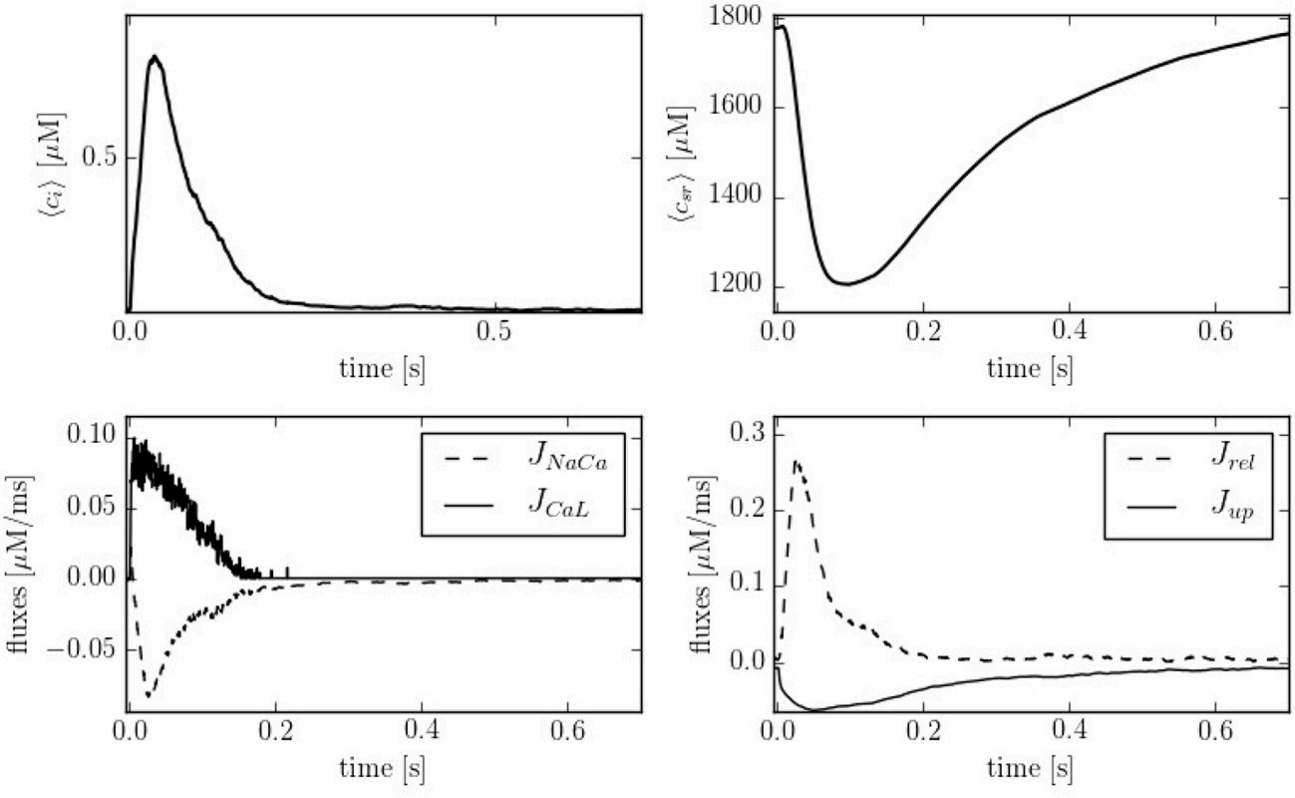
**(Top)** Temporal profiles of [Ca^2+^] for both domains, cytoplasm and SR. **(Bottom left)** Inward and outward cell currents of *J*_*NaCa*_ (dashed line) and *J*_*CaL*_ (solid line). **(Bottom right)** SERCA pump flux (solid line) and release flux (dashed line) in the cytoplasm at a pacing period of 800 ms.

Figure 4 shows typical traces during one beat of the average calcium over all the cell in both domains: cytoplasm and SR. The calcium peak, of ~700–800 nM, agrees well with experimental observations (Mackenzie et al., 2004). The calcium concentration in the SR, though, is larger than observed in experiments due to the lack of the SR buffer calsequestrin (CSQN) in our model. We also show in Figure 4 the four cytoplasmic fluxes, corresponding to the sodium-calcium exchanger (*J*_*NaCa*_), the L-type calcium flux (*J*_*CaL*_), SR release (*J*_*rel*_), and SERCA (*J*_*up*_). Due to the small number of LCC channels, the L-type calcium flux is particularly stochastic.

**Figure 5:**
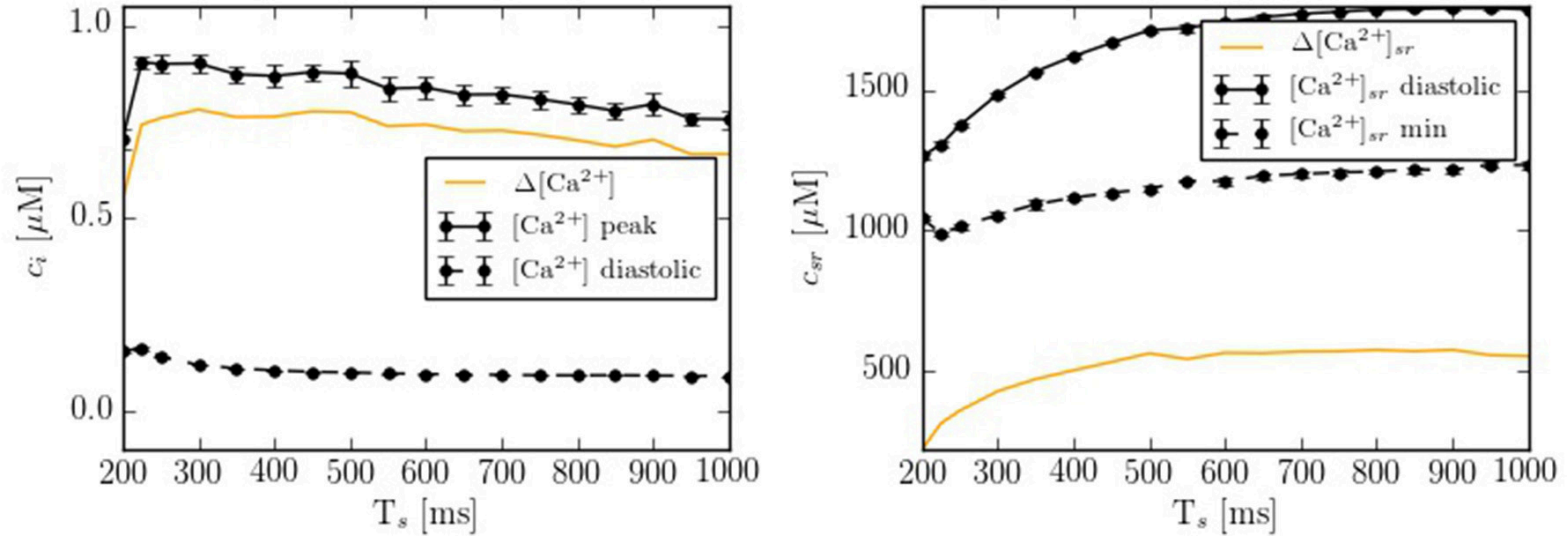
Ca^2+^ peak and Ca^2+^ basal level as function of the pacing period (*T*_*s*_) in both domains: cytoplasm and SR. Each point has been averaged over 10 beats in steady state.

Depending on the pacing period, the model shows different behaviors. We have quantified this effect by calculating the calcium peak and the calcium diastolic level in the cytoplasm and in the SR domain (Figure 5). To assure that the system is close to the steady state, we have paced the cell for 50 s at each pacing period, and then taken the average over the next 20 stimulations. As the pacing period decreases, the cytosolic calcium peak increases moderately, up to a pacing period period of ~200-300 ms, beyond which it decreases, due to the decrease in SR calcium content and fractional release. This behavior agrees qualitatively with the observed change in the contractile force as a function of pacing period observed in atrial cells (Maier et al., 2000; Schotten et al., 2002), that shows a peak at a period of ~500 ms, beyond which it decreases.

### 3.2. Inward Calcium Wave Propagation

**Figure 6:**
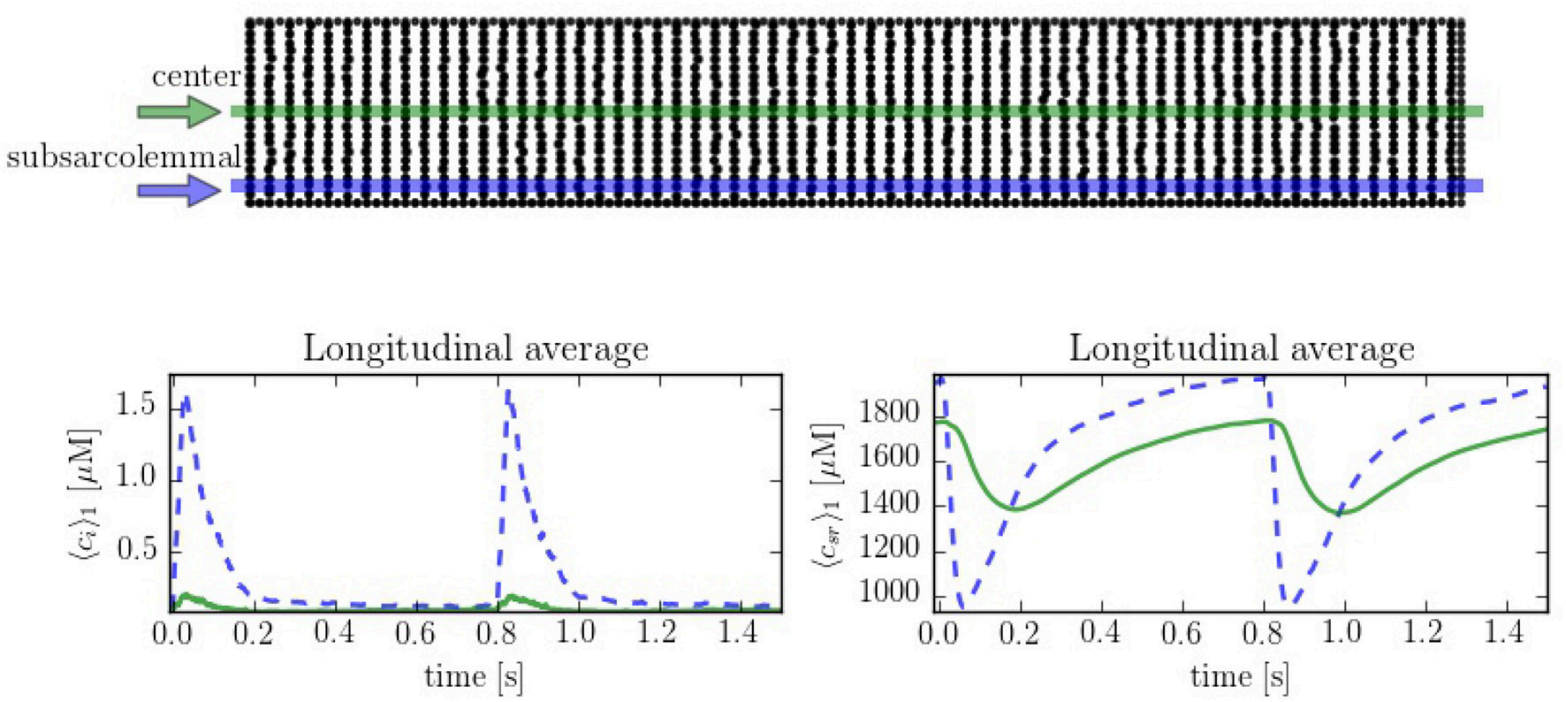
**(Top)** Spatial distribution of the CaRU. Black dots show the position of the CaRUs. The green and blue lines indicate longitudinal lines at the cell center and periphery. **(Down)** Local Ca^2+^ measured in the subsarcolemmal space (blue dashed lines) and the center of the cell (solid lines) for both cytoplasm and SR domains at a pacing period of 800 ms. All traces have been averaged over a longitudinal section of 1 μm width.

In order to compare the spatial heterogeneities within the cell, we have considered longitudinal sections at the central and peripheral regions, averaged over a 1 μm width. The complete CaRU distribution is shown in Figure 6, where the longitudinal sections are plotted in green and blue. Simulations suggest strong differences between calcium levels in the subsarcolemmal space and the center of the cell (see Figure 6), as well as a delay between release at the peripheral and central regions.

**Figure 7:**
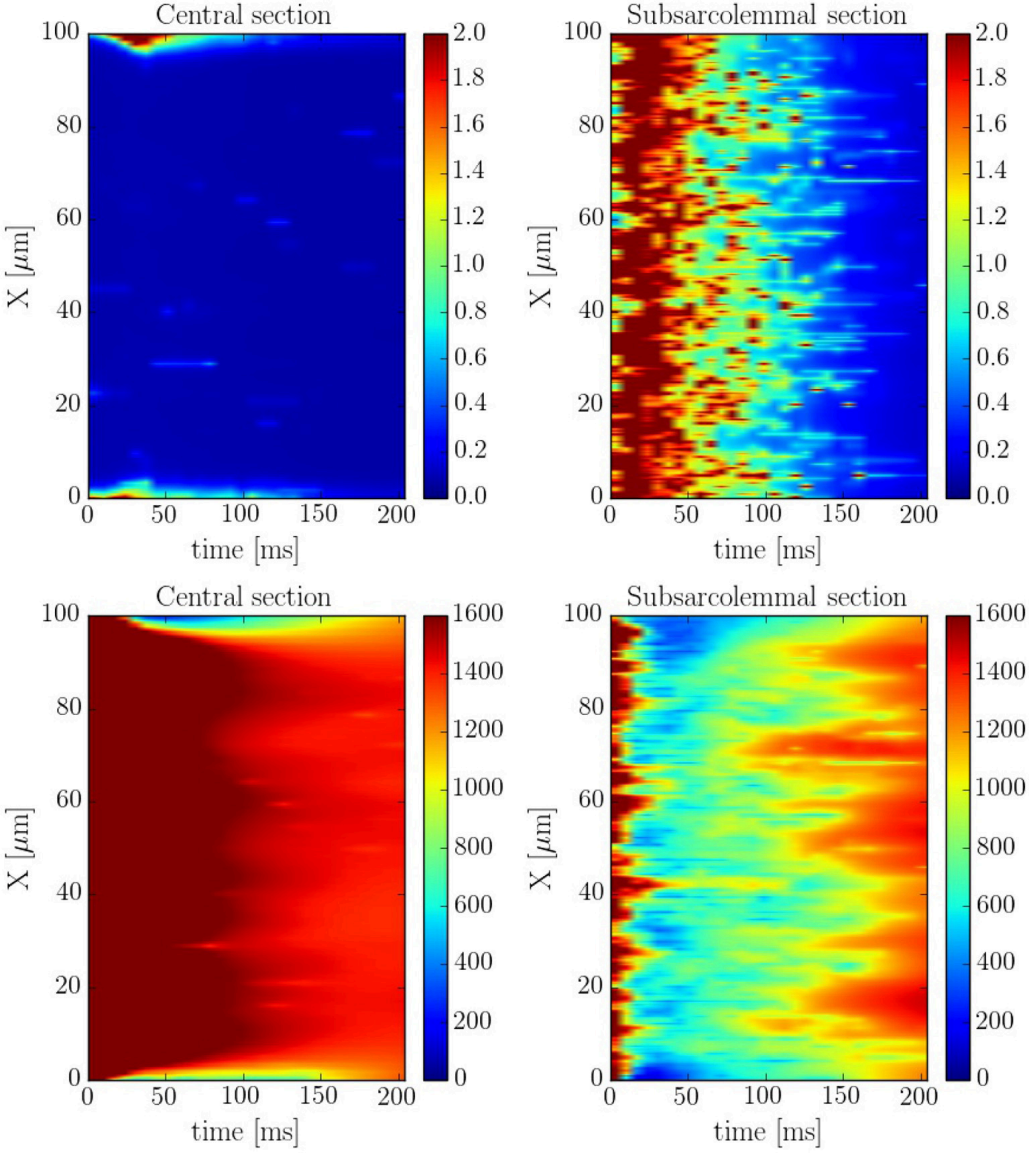
Longitudinal line scan during a single beat on the subsarcolemmal and the central region at a pacing period of 800 ms for cytosolic **(Top)** and SR **(Bottom)** calcium. The colorbar corresponds to calcium concentration in μM.

The spatio-temporal and local correlation between *c*_*i*_ and *c*_*sr*_ calcium is shown in the line scan profiles on Figure 7. The four profiles correspond to the same beat. In the subsarcolemmal region the presence of LCCs and CaRUs results in an important release activity causing a relevant SR depletion. On the other hand, in the central region, calcium does not penetrate, and the local activity is scarce. Still, there is a depletion of the SR content (visible also in Figure 6) due, not so much to release, almost negligible at the central region, but to diffusion of SR calcium to the periphery.

**Figure 8:**
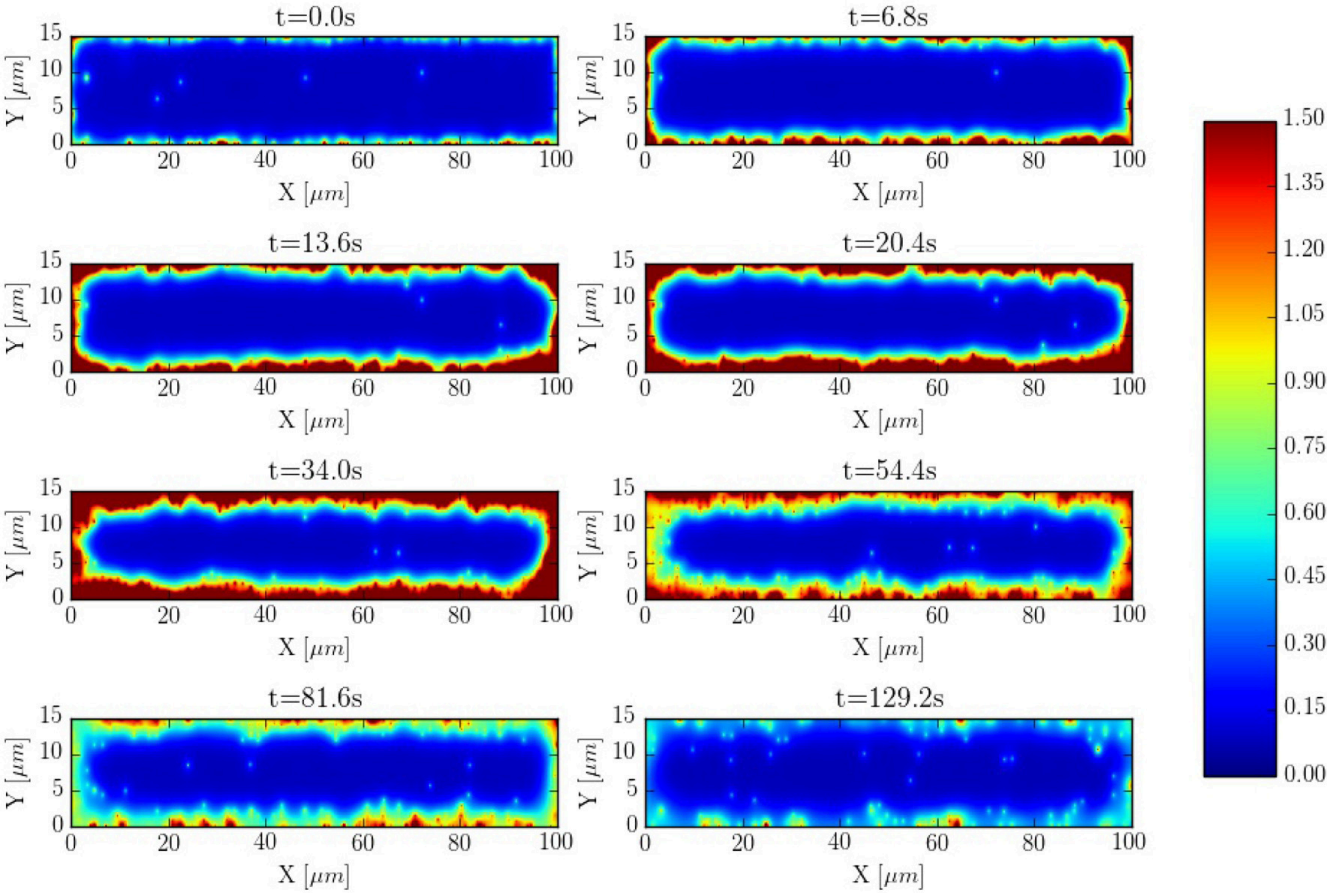
Inward wave propagation at a pacing period of 800 ms. The colorbar corresponds to calcium concentration in μM.

**Figure 9:**
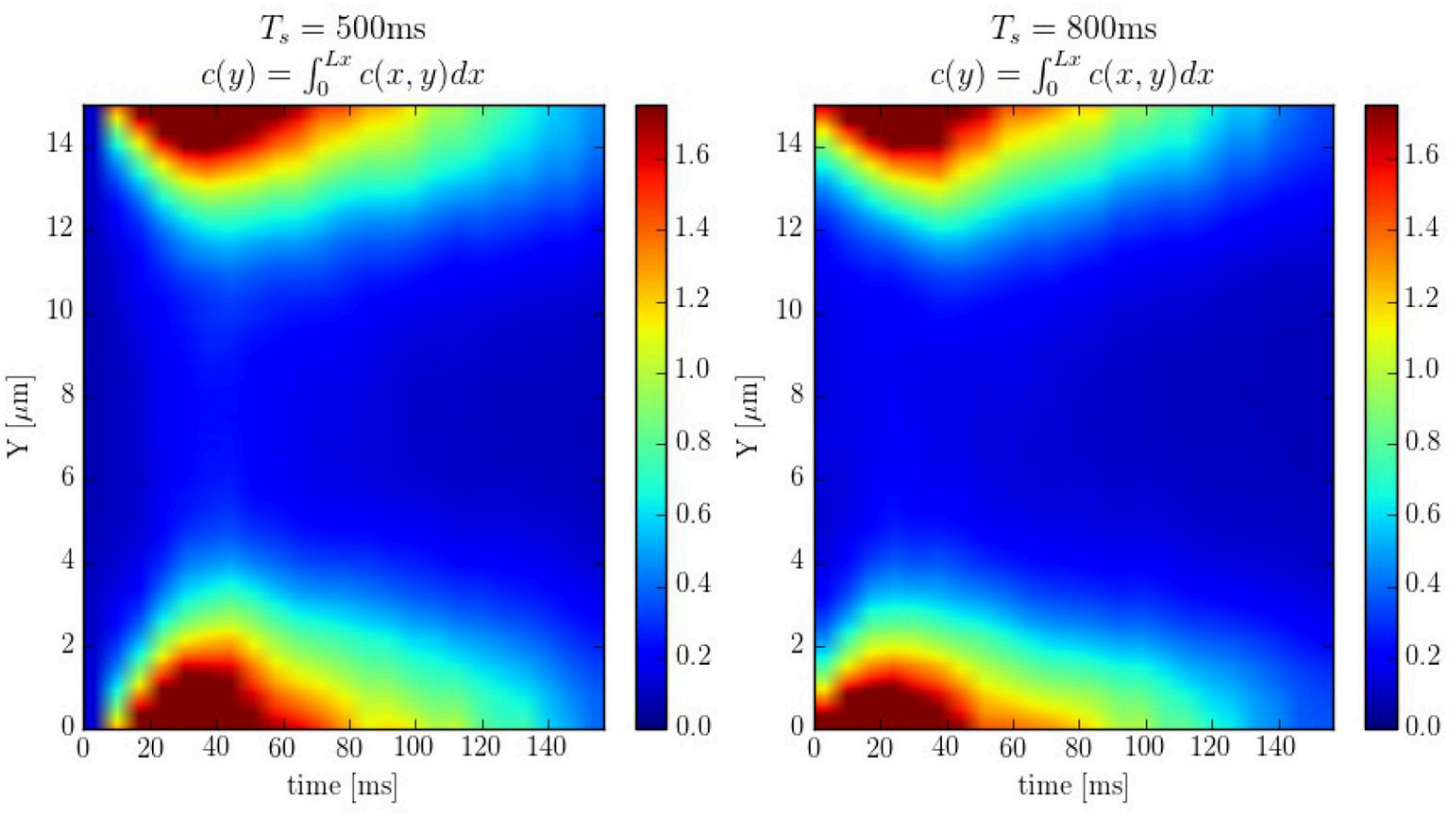
Line scans averaged over the longitudinal direction at pacing periods of 500 and 800ms. The colorbar corresponds to calcium concentration in μM.

The spatio-temporal Ca^2+^ dynamics in the cytoplasm allows us to clearly understand how the standard inward wave propagation occurs. Figure 8 shows spatial profiles at different times during a single beat. Under normal conditions, the calcium wave starts on the cell membrane and propagates to the center but this propagation does not reach the central region. This situation is observed more clearly averaging the calcium concentration over the longitudinal direction, so we can observe the average inward propagation of the calcium wave (Figure 9) Typically, the inward wave propagates 4 or 5 μm in the transversal direction. From the figure, we can estimate an inward wave velocity of roughly 150 μm/s, that agrees well with typical observed calcium wave velocities of ~100 μm/s (Izu et al., 2013).

**Figure 10:**
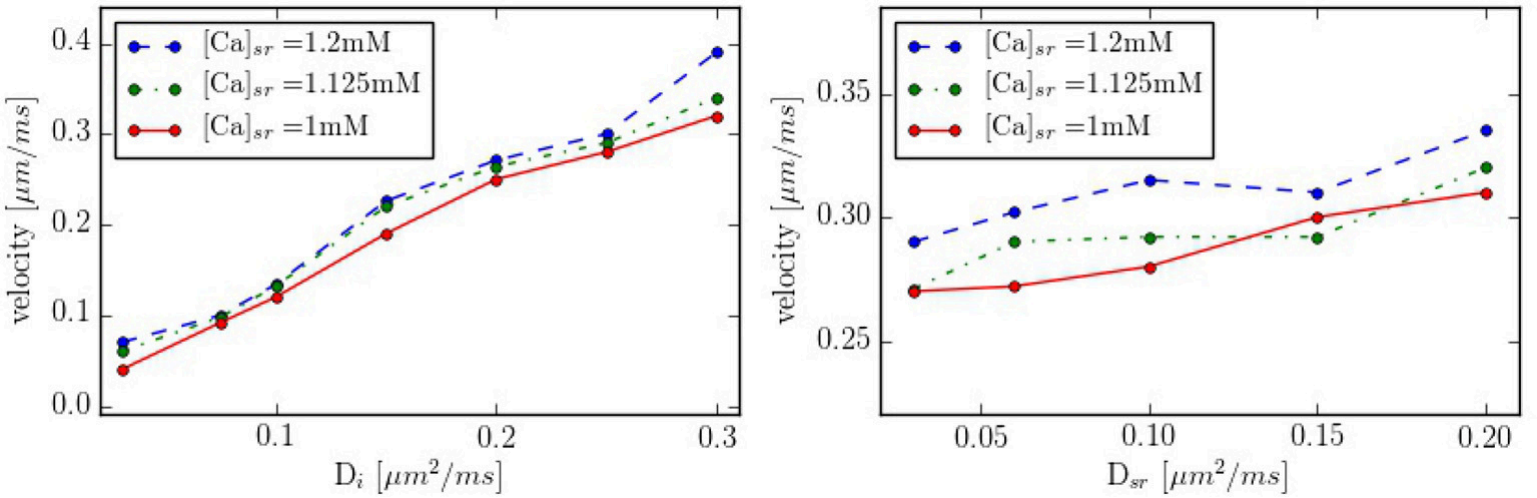
Propagation velocity as function of the diffusion constants *D*_*i*_ and *D*_*sr*_ for different values of the SR calcium load. As standard values we take *D*_*i*_ = 0.25 μm^2^/ms and *D*_*sr*_ = 0.09 μm^2^/ms in the zone between z-lines.

Intracellular waves are Ca^2+^ release events that propagate across the cell at a constant velocity. To have a better control of the calcium wave and be able to study its speed and dependence on different parameters, we have created a new geometry with 10 equidistant z-lines (see Figure S2, in Supplementary Material). The distance between two z-lines is 1.5 μm. Initially, cytosolic calcium at the first z-line is increased and then the system let to evolve without being forced. The wave front is monitored and the wave front velocity calculated. This way we determine the wave velocity as a function of different parameters. The typical wave velocity is of the order of 200−300 μm/s, that agrees well with a diffusive process within z-lines, that would give a speed of *v* ~ 2*D*/*d* ~ 2·200 μm^2^s^−1^/1.5 μm ~ 260 μm/s. This velocity increases slightly with the calcium SR load (Figure 10). The dependence on intracellular calcium diffusion *D*_*i*_ is roughly linear, as one would expect in saltatory dynamics (Dawson et al., 1999). The dependence on SR calcium diffusion *D*_*sr*_, on the other hand, is not so pronounced.

### 3.3. Effect of the Cell Structure

**Figure 11:**
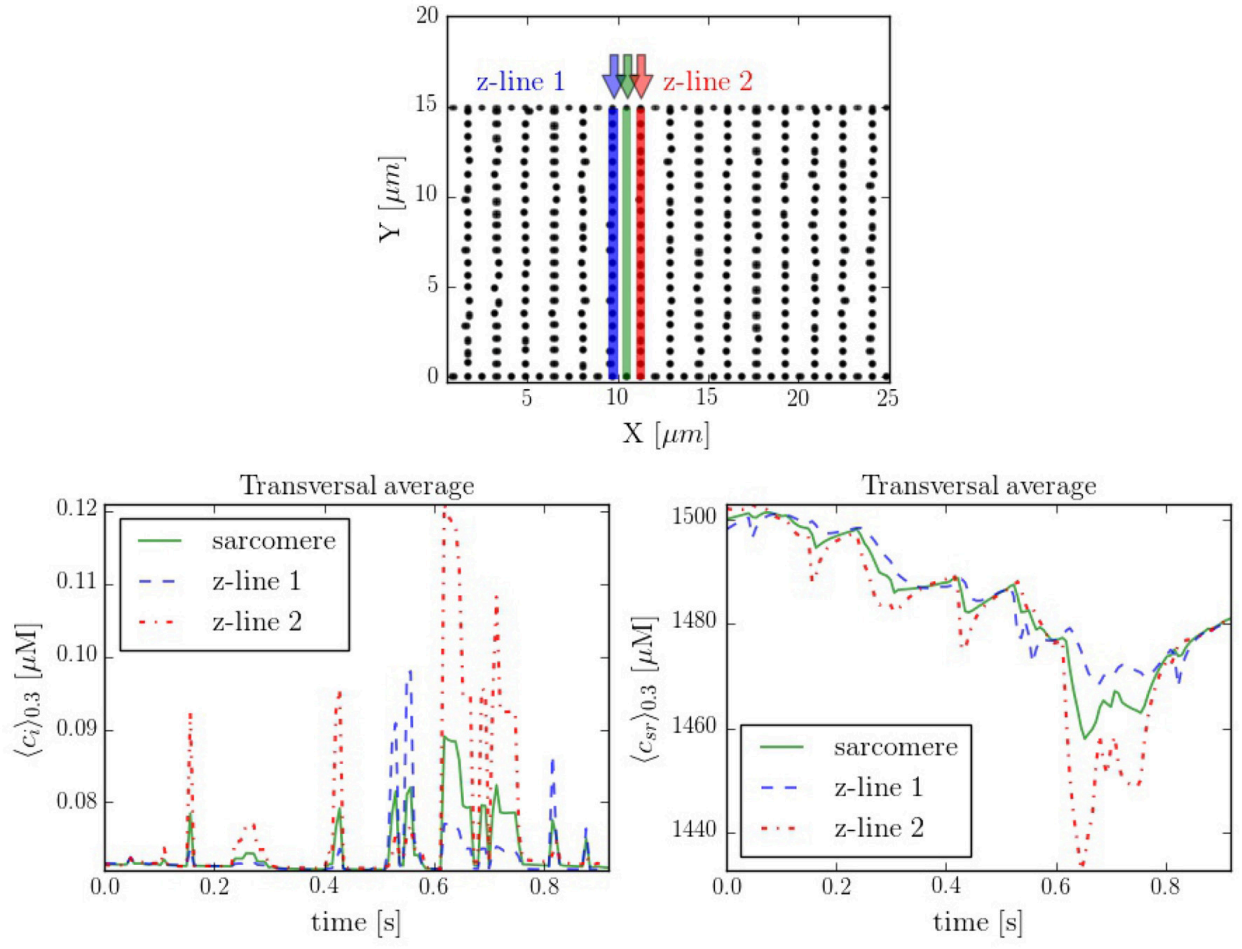
**(Top)** Spatial distribution of the CaRUs. Black dots show the position of the CaRUs. The blue and red lines indicate two transversal lines within neighboring z-lines. The green line a transversal line within those two z-lines. **(Down)** Local Ca^2+^ measured in the three transversal lines in post-rest potential conditions. All traces have been averaged over a transversal section of 0.3 μm width.

It is interesting to compare also the calcium dynamics at the z-lines and in the space within z-lines, where no CaRUs are present. To this end we have performed transversal section measurements, as shown in Figure 11, in a situation when the cell is at rest, without external stimulation. The sarcomere measurement corresponds to the space between the z-lines. It is important to notice that, because of the proximity between the measurements, there exists a correlation between the resulting profiles. For instance, at *t* = 0.6 s there is a spark that starts in the first z-line, it propagates to the sarcomere region and, then, to the second z-line. The second thing to notice is that, due to the presence of random Ca^2+^ releases associated to the position of the RyRs, at the z-lines the calcium trace is more stochastic (Figure 11).

### 3.4. RyR Cluster Structure and Distribution

**Figure 12:**
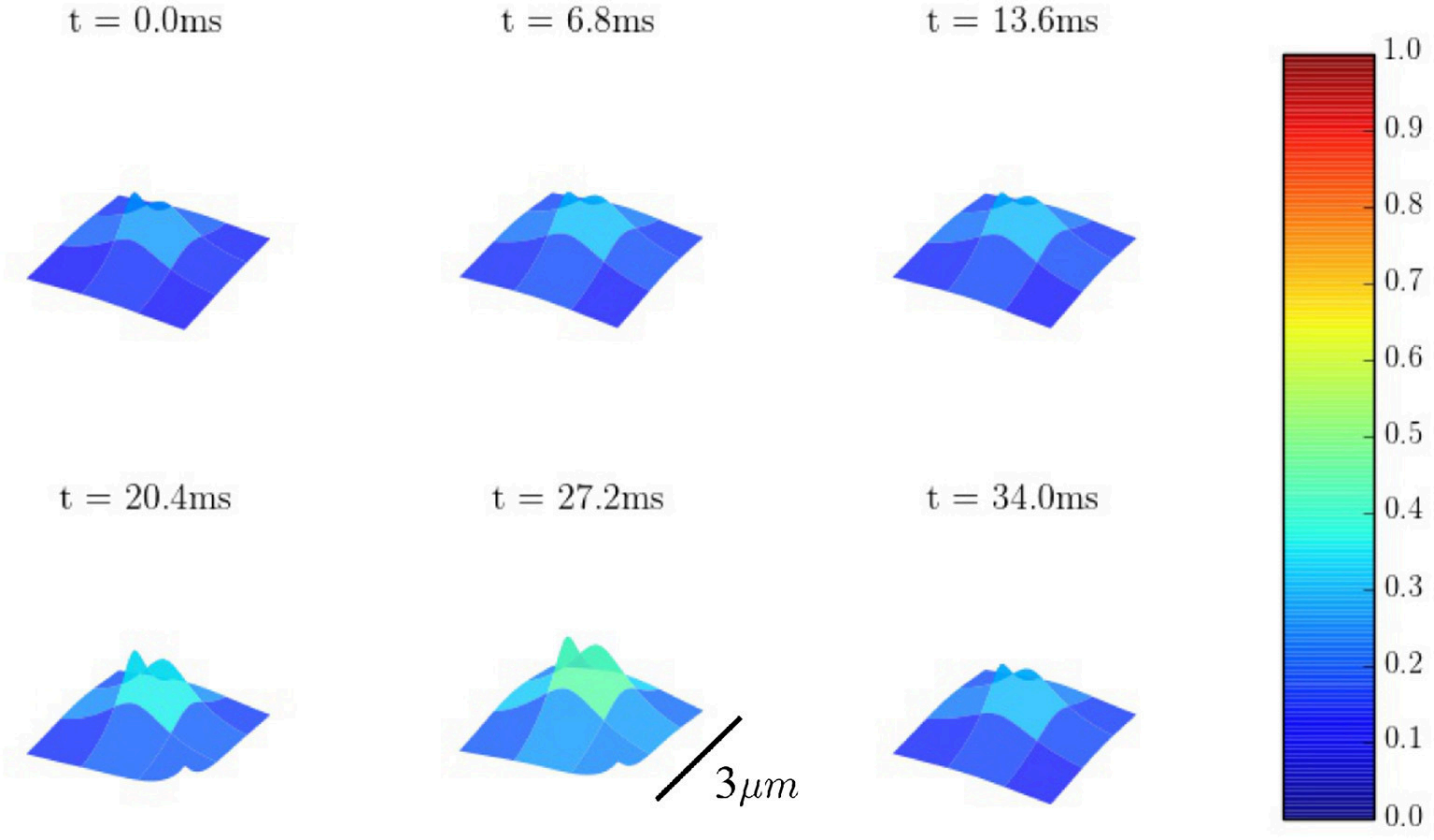
Spatial profiles of a Ca^2+^ spark.

**Figure 13:**
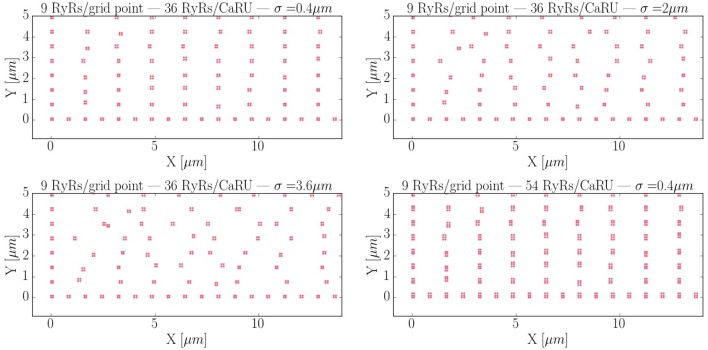
Different RyR distributions considered in the text. The bottom left corner of the whole cell is displayed. The red dots represent grid points with RyRs. First: control configuration, with standard dispersion in the position of the RyRs σ = 0.4 μm. Second: increased the dispersion of σ to 2 μm. Third: σ = 3.6 μm. Fourth: New structure configuration, where each grid point contains 9 RyRs and a CaRU is formed by 6 grid points, so that, each CaRU represents 54 RyRs. The total number of RyRs remains constant but now, in the new structure, they are more grouped, that is, the CaRUs are bigger. The dispersion is the same as in the standard case: σ = 0.4 μm.

Calcium sparks are the basic calcium release events. A good understanding of their characteristics (size, amplitude, and frequency) is thus very important to properly characterize the process of CICR. Due to the fine discretization of our model, we can observe their detailed spatio-temporal profiles. Figure 12 shows, for instance, the standard time evolution of a spark. We have also studied the effect of the microstructure in the frequency of sparks. We have modified the microstructure, changing the size and distribution of the CaRUs. This is particularly important since it has been observed that the RyR distribution changes in a particular way under conditions of AF (Macquaide et al., 2015). We have then calculated the spark frequency under resting conditions for different configurations defined by the Gaussian distribution of position sites and size of the CaRUs. For the standard size of the CaRU (36 RyRs divided equally among 4 grid points, each one containing 9 RyRs), we have considered three values of the dispersion in the Gaussian distribution, the standard value of σ = 0.4 μm, and two cases with larger dispersion of σ = 2 and 3.6 μm, see Figure 13. Besides, we also consider the effect of a change in the CaRU size, considering CaRUs with 54 RyRs, divided equally among 6 grid points, each one containing 9 RyRs (Figure 13 down right), but maintaining fixed the total number of RyRs in the cell. Since the total number of RyRs is the same, this means that there are larger, but less CaRUs in the cell.

**Figure 14:**
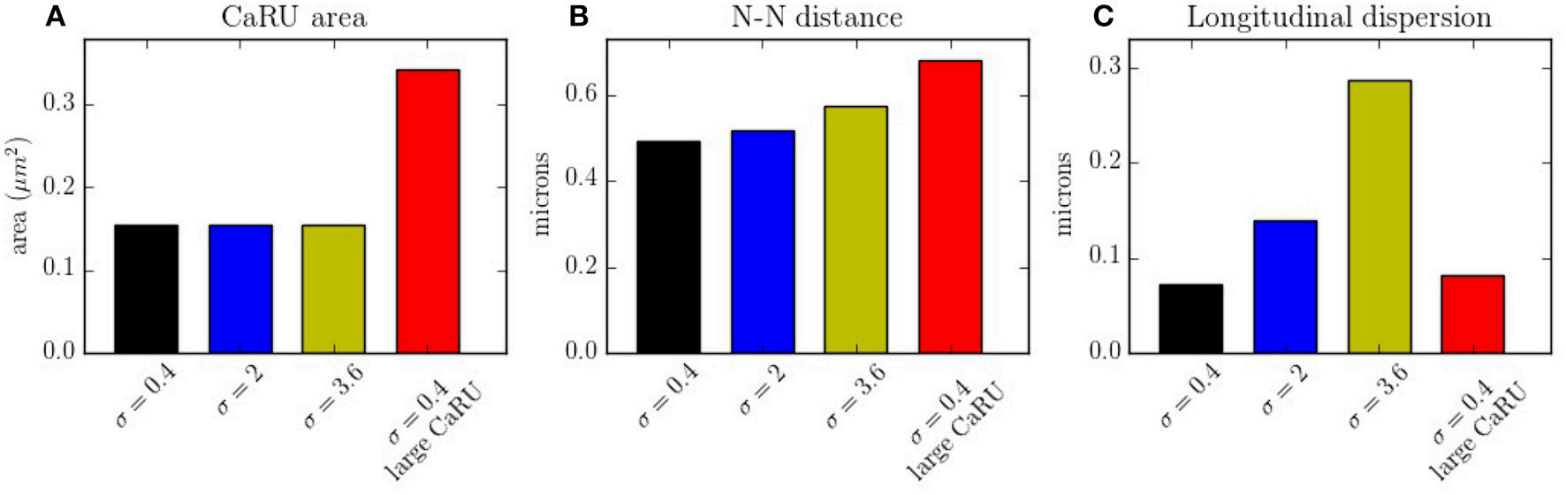
**(A)** Mean area occupied by a CaRU. **(B)** Nearest-neighbor distance among CaRUs, measured from the center of each CaRU. **(C)** Longitudinal dispersion, measured as the mean distance between the RyRs and the center of the z-line. All calculations have been averaged over 35 configurations.

In Figure 14A the average mean CaRU size is shown. To define the size of a CaRU, we follow the results by Macquaide et al. (2015), that showed using a computational model that clusters closer than 150 nm triggered together functionally as a single cluster. We thus assume that a group of clusters belong to the same CaRU if they are separated, at most, by 0.15 μm edge to edge from each other. By definition, there is a random component in the structure, so that, there is a non-zero dispersion on the distance with respect to the z-line (Figure 14C). Finally, the mean nearest neighbor distance is shown in Figure 14B. In the new structure, since the RyRs are more grouped, the total number of CaRUs is smaller, such that the mean distance among them increases.

**Figure 15:**
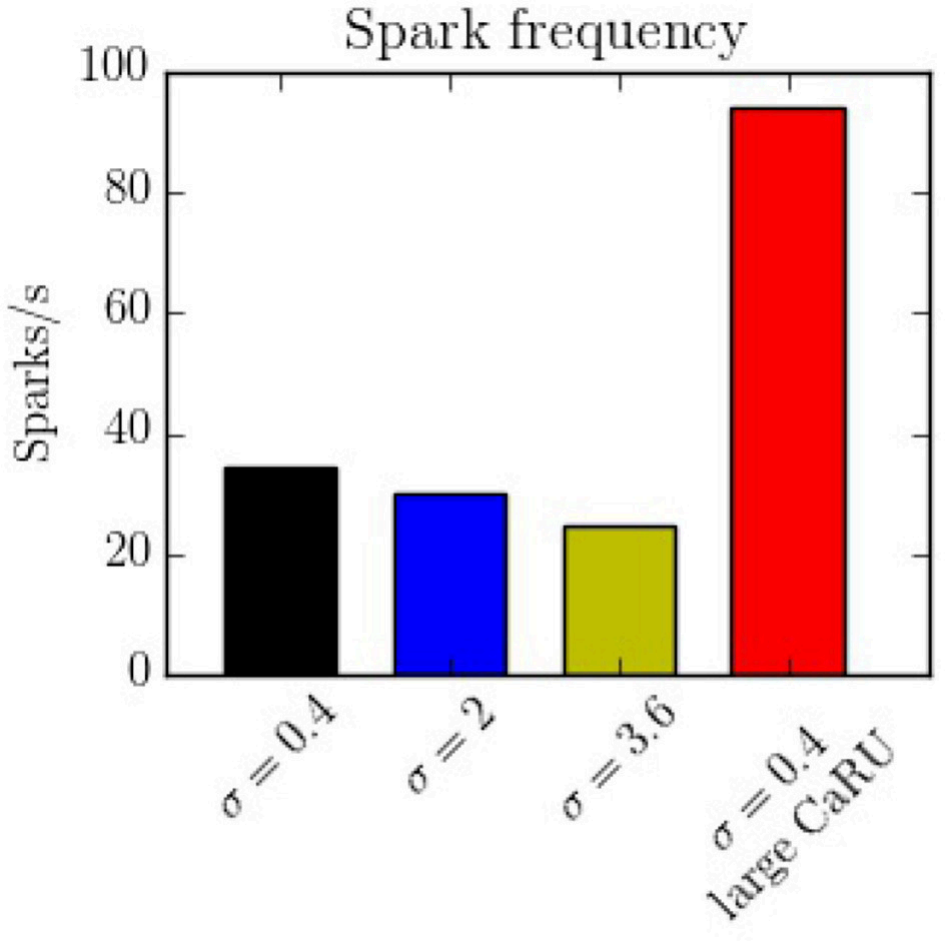
Spark frequency for the four different configurations. The spark frequency decreases with nearest-neighbor distance but increases with the size of the CaRU.

The spark frequency is one of the most important indicators of the stochastic activity during a post-rest potential period. The total number of spontaneous calcium sparks has been recorded. In order to consider the sparks, we have counted all the calcium release events greater than a certain spatial radial threshold of 1.6 μm. As shown in Figure 15, we observe that the frequency decreases with the longitudinal dispersion meaning that the cluster-cluster communication plays an important role in stochastic activity. On the other hand, when the CaRUs are bigger (structure configuration) this probability increases.

## 4. Discussion and Conclusions

In this work we present a novel model to fully simulate a 2D longitudinal plane of a cardiac atrial cell. By modeling the intracellular calcium dynamics and solving the spatio-temporal Ca^2+^ reaction-diffusion equations, both local and global behavior have been recorded. Because of the high spatial resolution, the model allows us to study in detail the dynamics in the surroundings of the CaRUs, that is, the local calcium concentrations and the spark activity. It is also well-suited to study the effect of changes in the spatial distribution and form of CaRUs. In this regard, important differences have been noticed using different spatial configurations of RyRs, showing that the resulting calcium dynamics is highly dependent on the spatial distribution. We observe, for instance, a decrease in spark frequency with CaRU spatial dispersion (Figure 15). This is correlated with an increase in CaRU nearest-neighbor distance, suggesting that cooperativity among local release events at nearby CaRUs could play an important role in the generation of sparks. In fact, sparks (or macrosparks) encompassing several CaRUs have also been observed experimentally (Kockskämper et al., 2001). When the same total number of RyRs of the cell are distributed in larger CaRUs, we observe an increase in spark frequency (Figure 15). These larger CaRUs are obtained increasing their size, but maintaining the density of RyRs, similar to what is observed in atrial cells presenting AF (Macquaide et al., 2015). Due to the larger number of RyRs per CaRU, an increase in the spark probability of each individual CaRU is expected, but, since the number of CaRUs is decreased, the increase in spark frequency per CaRU must be non-linear with size. In this case, the increase in nearest-neighbor distance (Figure 14) does not result in a decrease in spark rate. However, it should be noted that we have measured the nearest-neighbor distance from the centers of the CaRUs, but since their size is larger, the distance between the edges of the CaRUs could actually be similar, or smaller. A more detailed study of the influence of the CaRU structure in spark frequency, the appearance of macrosparks, and the transition to waves is deferred to a future study.

In our simulations, the opening of the L-type calcium channels induce a calcium increase in the periphery of the cell, that hardly reaches the interior (Figure 6). This result agrees with what is observed in atrial cells without t-tubules where, under basal conditions, calcium signals are restricted to subsarcolemmal regions (Mackenzie et al., 2004). The observed values of the calcium transients, slightly over 1 μM at the periphery and 0.2–0.3 μM at the center are similar to what we obtain in our simulations (Figure 6). As observed in previous works (Dawson et al., 1999), the speed of the wave varies roughly linearly with the diffusion coefficient in the cytosol. Although we have not changed the distance among z-planes in this work, the fact that this linear relation continues at low values of the diffusion constant, seems to indicate the lack of a threshold for propagation in the model. This contrasts with results by Izu et al. (2006) and Hoang-Trong et al. (2015) that found a strong dependence on wave initiation with the distance among CaRUs. However, one should notice that, for the calculation of wave propagation (Figure 10), we have considered an increased strength of the L-type calcium current, probably pushing the threshold to smaller values of the diffusion constant than we have considered. In our simulations, calcium waves are obtained at transients larger than in experiments and with diffusion and RyR cluster spacing in the upper and lower ranges, respectively, allowing for diffuse and fire behavior. However, one should mention the difficulty to reconciliate calcium wave propagation at low calcium concentrations with a stochastic description of the RyR cluster (Izu et al., 2013). Recent studies suggest also an important role of SR calcium diffusion for the propagation of the calcium wave, through junctional SR calcium depletion and sensitization of the RyRs (Keller et al., 2007). We find that the dependence with diffusion in the SR is not so pronounced (Figure 10), seemingly ruling out an important role of SR Ca diffusion in wave propagation. This effect, however, should be studied in more detail, as well as the role of buffers, RyR sensitivity and the level of cytosolic calcium in calcium wave propagation.

The present study presents several limitations. To cite some, that we consider a two-dimensional geometry, a voltage clamp protocol, isotropic diffusion, or immobile buffers. The main reason to use a two-dimensional geometry was computational cost. A generalization to three-dimensions is straight-forward and we are implementing it to study some of the questions posed in this article in more detail. The dynamics of transmembrane voltage can be also readily incorporated into the model, and could be used to study the arrhythmogenic effect of spontaneous calcium release events, for instance. The correct characterization of calcium diffusion in the cell represents a harder challenge. We have considered typical values of the diffusion coefficients in the cytosol and SR and assumed that they depend linearly on the cytosolic/SR volume fractions, as suggested by homogenization (Goel et al., 2006). However, diffusion (particularly in the SR) is most likely to be anisotropic, and this could importantly affect wave characteristics. A better knowledge of the SR microstructure could help to estimate these diffusion coefficients and give a better representation of calcium wave propagation. An important addition would be to incorporate a (partial) network of t-tubules. The presence of t-tubules in atrial cells has been found to depend on the species, and there is evidence of their presence in large mammals' atrial cells (Richards et al., 2011). Besides transversal, axial tubules have also been found to contribute to rapid activation of the atrial cell (Brandenburg et al., 2016). In heart disease, including human heart failure (HF), there is extensive remodeling, resulting in loss and disorganization of t-tubules (Dibb et al., 2013). Besides, there are other important factors that may affect calcium transients. For instance, the presence of IP_3_R may affect the form of Ca^2+^ sparks, leading to a difference between calcium handling at the peripheral and central regions (Mackenzie et al., 2004; Kim et al., 2010). Another important effect, not included in our model, is the presence of mitochondria. In venticular myocytes, there is evidence suggesting that the mitochondrial outer membrane is linked to t-tubules (Hayashi et al., 2009). Models of excitation-contraction coupling, including mitochondrial calcium handling have been developed for ventricular myocytes (Cortassa et al., 2003, 2006; Matsuoka et al., 2004; Maack and O'rourke, 2007; Hatano et al., 2011) and used, to study, for instance, the influence of the distance between mitochondria and Ca^2+^ release sites (Hatano et al., 2013). In the atria, the mitochondria has been suggested to act as a buffer that prevents inward calcium propagation (Mackenzie et al., 2004). The effect of these structural factors on wave propagation is an important matter for future work.

## Author Contributions

MM assisted with the design of the work, performed the simulations, analyzed the data and prepared the manuscript. BE obtained funds, designed the work and revised and edited the manuscript. All authors approved the final manuscript.

### Conflict of Interest Statement

The authors declare that the research was conducted in the absence of any commercial or financial relationships that could be construed as a potential conflict of interest.
