# Supplementary material for: Computational Model of Calcium Signaling in Cardiac Atrial Cells at the Submicron Scale
Source: Front Physiol. 2018 Dec 10;9:1760. doi: 10.3389/fphys.2018.01760 (PMC6295473; doi:10.3389/fphys.2018.01760)
Supplement: Supplementary file 1 [file Data_Sheet_1.pdf]

Supplementary Material: Computational model of calcium signaling in cardiac atrial cells at the submicron scale

1 POTENTIAL

The transmembrane potential is the external stimulus that triggers calcium cycling. We will take the voltage clamp approximation, where the time variation of the transmembrane potential is fixed and is defined based on Shiferaw et al. (2003); Weiss et al. (2006):

$$V(t) = \begin{cases} (V_{max} - V_{rest})\sqrt{1-(\tilde{t}/APD)^2} + V_{rest}, & \tilde{t} < APD \\ V_{rest}, & \tilde{t} > APD \end{cases} \tag{S1}$$

where $\tilde{t} = t - nT_s$, with $n = 0, 1, 2, \ldots$, T_s is the stimulus period and the action potential duration (APD) is a function of that period (in ms) given by: $APD = 100T_s/(100 + T_s)$.

2 MODEL DESCRIPTION

In section II of the main manuscript we have presented the principal equations that govern our system. Just to give more detail, the expression of currents J_i and J_{sr} used can to be split up in cases. In particular, for the already defined regions (Ω_c, Ω_{sr} and $\delta\Omega$) we have that:

- Inter z-planes space ($\mathbf{r} \in \Omega_c$):

$$J_i(\mathbf{r}, t) = -J_{sr}(\mathbf{r}, t) = -J_{up}(\mathbf{r}, t) \tag{S2}$$

  These currents are defined in internal points without RyR.
- Internal cytosolic space along the z-lines ($\mathbf{r} \in \Omega_{sr} \setminus \delta\Omega$):
  - without RyR:

$$J_i(\mathbf{r}, t) = -J_{sr}(\mathbf{r}, t) = -J_{up}(\mathbf{r}, t) \tag{S3}$$

  - with RyR:

$$J_i(\mathbf{r}, t) = -J_{sr}(\mathbf{r}, t) = J_{rel}(\mathbf{r}, t) - J_{up}(\mathbf{r}, t) \tag{S4}$$

- Submembrane space ($\mathbf{r} \in \delta\Omega$):
  - without RyR:

$$J_i(\mathbf{r}, t) = J_{NaCa}(\mathbf{r}, t) - J_{up}(\mathbf{r}, t), \tag{S5}$$

$$J_{sr}(\mathbf{r}, t) = J_{up}(\mathbf{r}, t) \tag{S6}$$

- with RyR (so, with LCC):

$$J_i(\mathbf{r},t) = J_{NaCa}(\mathbf{r},t) + J_{rel}(\mathbf{r},t) - J_{CaL}(\mathbf{r},t) - J_{up}(\mathbf{r},t), \tag{S7}$$

$$J_{sr}(\mathbf{r},t) = J_{up}(\mathbf{r},t) - J_{rel}(\mathbf{r},t). \tag{S8}$$

In the following section we will give a broad description of the currents used above.

3 ELECTROPHYSIOLOGICAL CURRENTS

3.1 Release current

The transition dynamics between each state (in each cluster) is solved stochastically using a time-adaptive Gillespie's method Nivala et al. (2012). Cooperativity in activating Ca^{2+} binding is incorporated by transition rates depending on c_i^2. In particular, four transition rates are calcium dependent (see Fig. 3):

$$k_{co}(c_i) = k_a c_i^2 \qquad k_{i2i1}(c_i) = k_b c_i^2 \qquad k_{oi} = k_c c_i \qquad k_{ci} = k_c c_i \tag{S9}$$

The other rates are constant with the following properties $k_{oc} = k_{i1i2}$, $k_{oi} = k_{ci}$. Detailed balance implies $k_{io} = k_{ic}(k_a/k_b)$. A lumenal SR calcium dependence has been implemented as in Shannon et al. (2004). The lumenal dependence is set as in Shannon et al. (2004):

$$k_{CaSR} = Max_{SR} - \frac{Max_{SR} - Min_{SR}}{1 + (EC_{50-SR}/Ca_{SR})^H_{SR}} \tag{S10}$$

3.2 SERCA current

The associated current to the SERCA pump is considered to be:

$$J_{up} = g_{up}\frac{(c_i/K_i)^2 - (c_{sr}/K_{sr})^2}{1 + (c_i/K_i)^2 + (c_{sr}/K_{sr})^2}. \tag{S11}$$

J_{up} can be outward or inward depending on the relative concentrations. We say that J_{up} is an inward flux when it increases the concentration in the SR. Otherwise, it will be outward. Since the denominator is always positive, an inward flux is achieved when:

$$\left(\frac{c_i}{K_i}\right)^2 - \left(\frac{c_{sr}}{K_{sr}}\right)^2 > 0 \rightarrow \frac{c_i}{K_i} > \frac{c_{sr}}{K_{sr}}, \tag{S12}$$

where K_i and K_{sr} represent the local equilibrium concentration of cytoplasmic and SR, respectively. Since we are considering an homogenized model, the SERCA pump is set in the whole cell.

3.3 Sodium-calcium exchanger

The Na-Ca exchanger is given by

$$J_{NaCa} = \frac{g_{NaCa}}{1 + (K_{da}/c_i)^3}\frac{e^{\eta z}[Na]_i^3[Ca]_0 - e^{(\eta-1)z}[Na]_0^3 c_i}{S(c_i)(1 + k_{sat}e^{(\eta-1)z})}, \tag{S13}$$

with $z = VF/(RT)$, V the membrane potential, F the Faraday constant, R the constant of gases and T the temperature. It depends on extracellular calcium and sodium concentrations $[Na]_0$, $[Ca]_0$, and on intracellular sodium $[Na]_i$, which we take as a parameter. The function $S(c_i)$ reads as:

$$\begin{aligned} S(c_i) =& [Na]_0^3 c_i + K_{mNa0}^3 c_i [1 + (c_i/K_{mCai})] \\ &+ K_{mCa0}[Na]_i^3 + [Na]_i^3 [Ca]_0 \\ &+ K_{mCai}[Na]_0^3 [1 + ([Na]_i/K_{mNai})^3]. \end{aligned}$$

The pump will arrive at the equilibrium in a time scale controlled by the value of K_{da}. In that case, the equilibrium concentrations for the exchanger depend on the transmembrane voltage and is given by:

$$c_i^{eq} = \frac{[Na]_i^3 [Ca]_0}{[Na]_0^3} e^z. \tag{S14}$$

This pump will take place in all the cell membrane.

3.4 L-type Calcium current

The voltage dependent rates that determine the LCC dynamics (see Fig. 3) are given by:

$$\begin{aligned} & a_{12}(V) = p_\infty, \quad a_{21}(V) = 1 - p_\infty, \quad p_\infty = \frac{1}{1 + e^{-(V-15)/8}}, \\ & a_{15}(V) = p_0/\tau_0, \quad a_{51}(V) = (1 - p_0)/\tau_0, \\ & p_0 = \frac{1}{1 + e^{-(V+40)/10}}, \quad \tau_0 = (\rho_0 - 450)p_{0f} + 450, \\ & \rho_0 = 10 + 4954 e^{V/15.6}, \quad p_{0f} = 1 - \frac{1}{1 + e^{-(V+40)/4}}, \\ & a_{45}(V) = (1 - p_{if})/3, \quad p_{if} = \frac{1}{1 + e^{-(V+40)/3}}. \end{aligned} \tag{S15}$$

Notice that these rates depend on voltage. When the membrane depolarizes, that is $V \sim 40$mV, the LCC goes to the open state. Moreover, there are rates dependent on cytoplasmic calcium concentration:

$$\begin{aligned} & a_{24} = 0.00413 + 0.024 f_{ca}, \quad a_{34} = 0.00195 + 0.01826 f_{ca}, \\ & \text{with } f_{ca} = \frac{1}{1 + (K_{LCC}/c_i)^3}, \end{aligned} \tag{S16}$$

providing the inactivation of the LCC. Detailed balance also requires:

$$\begin{aligned} & a_{43} = a_{34}(a_{23}/a_{32})(a_{42}/a_{24}), \\ & a_{54} = a_{45}(a_{51}/a_{15})(a_{24}/a_{42})(a_{12}/a_{21}) \end{aligned} \tag{S17}$$

This current will appear in those points which present a LCC group (all of them located in the cell membrane).

3.5 Buffer dynamics

The dynamics for each of the three buffer concentrations ($c_{b,j}$) is given by

$$J_{b,j} = k_{on,j} c_i (B_T - c_{b,j}) - k_{off,j} c_{b,j} \tag{S18}$$

with j representing TnC, CaM or SR buffers.

4 DIFFUSION COEFFICIENT

Since the problem has two different domains (cytoplasm and SR), in general we have to set two different diffusion coefficients. We are working in a homogenized model, so that, calcium could diffuse over all domain. In order to take into account geometrical considerations (as we know, SR forms a complex branching network), we set effective diffusion coefficients that depend on the volume fraction v_i/v_{sr} Goel et al. (2006). This dependence is plotted in Fig. S1.

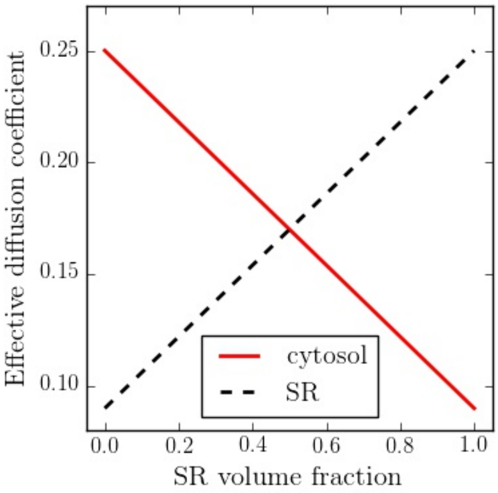

Figure S1: Variation of the effective diffusion coefficient (in units, μm^2/s) with the SR volume fraction. It is a linear approximation of the result obtained in Goel et al. (2006).

5 WAVE PROPAGATION MODEL

In order to have a better measure of the wave speed, we have developed a toy model with a simplified CaRU distribution. The spatial distribution of CaRUs is shown in Fig. S2.

This basic toy model allows us to study how propagation proceeds from plane to plane. The simulation starts with a stimulus at the leftmost z-line leading to the firing of the first z-line. Then, the wavefront diffuses to the second z-line that also fires. This process causes a macroscopic wave that propagates from one side of the domain to the other. Fig. S3 shows spatial profiles at different times during the propagation. Given that, the mean penetration distance of the wavefront is plotted in Fig. S4. From that, the mean wavefront velocity is calculated.

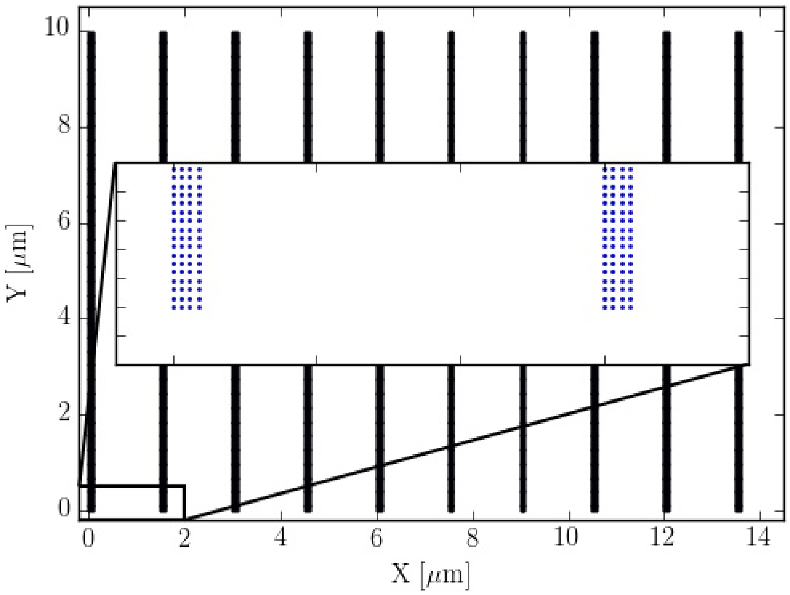

Figure S2: CaRU distribution.

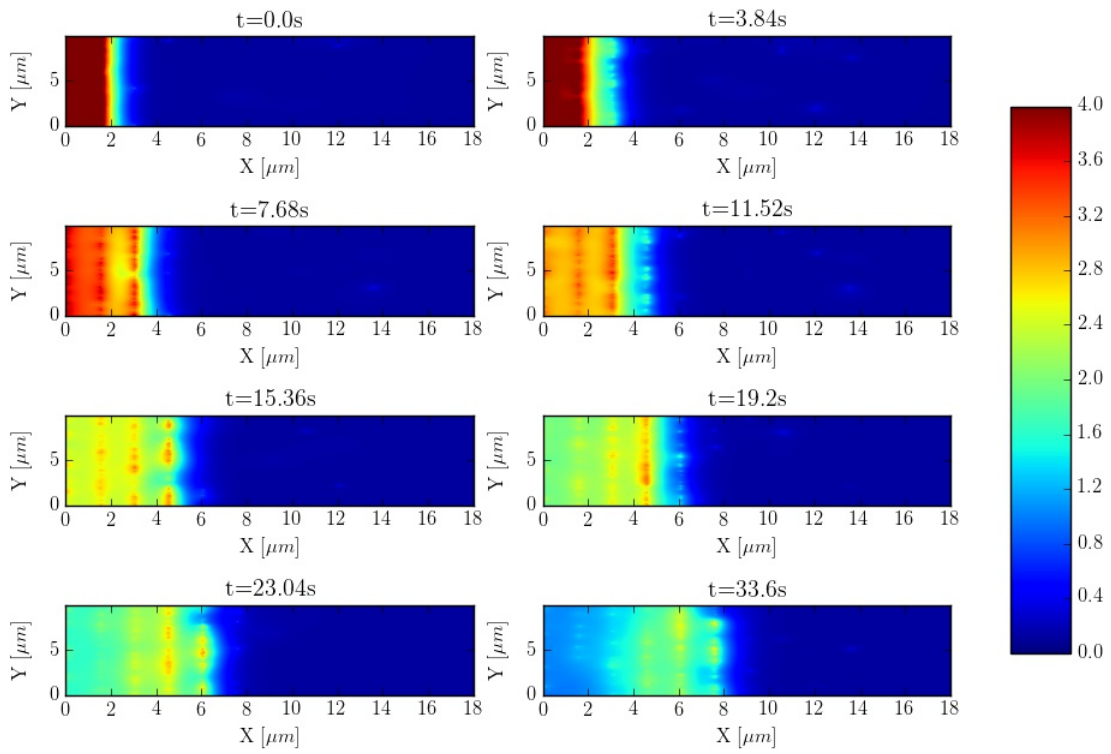

Figure S3: Calcium wave propagation.

6 SUPPLEMENTARY TABLES AND FIGURES

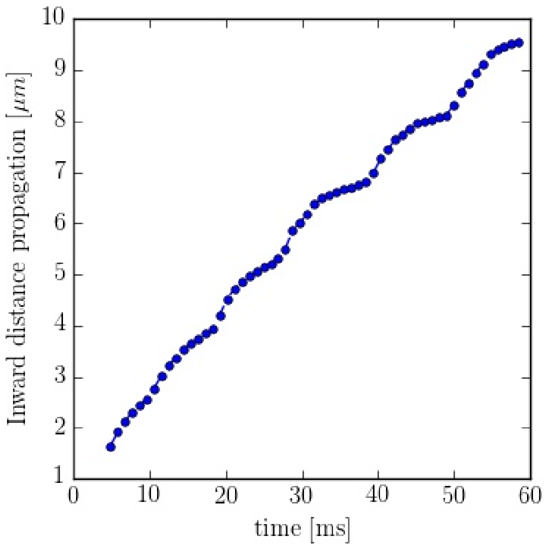

Figure S4: Inward distance propagation.

REFERENCES

Shiferaw Y, Watanabe M, Garfinkel A, Weiss J, Karma A. Model of intracellular calcium cycling in ventricular myocytes. *Biophysical journal* **85** (2003) 3666–3686.

Weiss JN, Karma A, Shiferaw Y, Chen PS, Garfinkel A, Qu Z. From pulsus to pulseless the saga of cardiac alternans. *Circulation research* **98** (2006) 1244–1253.

Nivala J, Knowles P, Dotro G, García J, Wallace S. Clogging in subsurface-flow treatment wetlands: measurement, modeling and management. *Water research* **46** (2012) 1625–1640.

Shannon TR, Wang F, Puglisi J, Weber C, Bers DM. A mathematical treatment of integrated ca dynamics within the ventricular myocyte. *Biophysical journal* **87** (2004) 3351–3371.

Goel P, Sneyd J, Friedman A. Homogenization of the cell cytoplasm: the calcium bidomain equations. *Multiscale Modeling & Simulation* **5** (2006) 1045–1062.

Table S1. Parameters.

**General parameters**		
time step	Δt (ms)	
spatial step	Δx (μm)	0.1
Resting potential	V_{res} (mV)	-85
Maximum potential	V_{max} (mV)	10
cell x-length	L_x (μm)	100
cell y-length	L_y (μm)	15
volume factor (cytoplasm)	v_i/v_{sr}	200
volume factor (SR)	v_i/v_{sr}	20
volume myocyte	v_{myo} (μm^3)	21500
Temperature	T(K)	308
Faraday constant	F(C/mol)	96485
Ideal gas constant	R(J/(Kmol))	8.31
**Buffer Parameters**		
TnC concentration	B_{TnC}(μM)	70
TnC binding rate	$k_{on,TnC}$(μM^{-1}ms^{-1})	0.00327
TnC unbinding rate	$k_{off,TnC}$(μM^{-1})	0.00196
Calmodulin concentration	B_{CaM}(μM)	24
Calmodulin binding rate	$k_{on,CaM}$(μM^{-1}ms^{-1})	0.003
Calmodulin unbinding rate	$k_{off,CaM}$(μM^{-1})	0.02
SR-bound buffer concentration	B_{SR}(μM)	23.5
SR-bound buffer binding rate	$k_{on,SR}$(μM^{-1}ms^{-1})	0.01
SR-bound buffer unbinding rate	$k_{off,SR}$(μM^{-1})	0.006
**Na-Ca exchanger**		
Uptake strength exchanger	g_{NaCa}(μM/ms)	56
Extra cellular Ca concentration	$[Ca]_o$(mM)	1.8
Extra cellular Na concentration	$[Na]_o$(mM)	136
Intra cellular Na concentration	$[Na]_i$(mM)	10
Inactivation constant	K_{da}(μM)	0.275
Saturation constant	k_{sat}	0.27
Voltage sensitivity constant	η	0.35
External sensitivity constant for Na	K_{mNao} (mM)	87.5
External sensitivity constant for Ca	K_{mCao} (mM)	1.3
Internal sensitivity constant for Na	K_{mNai} (mM)	12.3
Internal sensitivity constant for Ca	K_{mCai} (mM)	0.0036
**SERCA parameters**		
Maximum uptake SERCA	g_{up}(μMms^{-1})	0.09
Half occupation of cytosolic calcium binding states	K_i(μM)	0.0615
Half occupation of SR calcium binding states	K_{sr}(mM)	1.3

Table S2. Parameters.

**General parameters**		
**RyR parameters**		
Single channel strength	g_{rel}(ms^{-1})	4
Number of RyR per CaRU	N_{RyR}	4-9
Closing rate	$k_{oc} = k_{i1i2}$(ms^{-1})	0.08
Inactivation rate	$k_{oi} = k_{ci}$(ms^{-1})	0.001
Recovery rate from $I \to C$	k_{ic}(ms^{-1})	0.0025
Opening rate parameter	k_a(μM^{-2}ms^{-1})	1.77 $\cdot 10^{-4}$
Transition in I states	k_b(μM^{-2}ms^{-1})	10^{-4}
Maximum value of k_{CaSR}	Max_{SR}	15
Minimum value of k_{CaSR}	Min_{SR}	1
Exponent in SR calcium lumenal dependence	H	5
Threshold concentration of SR calcium	EC_{50-SR}(μM)	350
**LCC parameters**		
Strength effective flux	g_{CaL} (μm^3/ms)	32610
Number of LCC per CaRU	N_{LCC}	5
Threshold Ca-induced transition	K_{LCC}(μM)	5
